# Dependence of Electronic and Optical Properties of MoS_2_ Multilayers on the Interlayer Coupling and Van Hove Singularity

**DOI:** 10.1186/s11671-019-3105-9

**Published:** 2019-08-19

**Authors:** Jia-Qi Hu, Xiao-Hong Shi, Shun-Qing Wu, Kai-Ming Ho, Zi-Zhong Zhu

**Affiliations:** 1 Department of Physics, OSED, Key Laboratory of Low Dimensional Condensed Matter Physics (Department of Education of Fujian Province), Jiujiang Research Institute, Xiamen University, Xiamen, 361005 China; 2 International Center for Quantum Design of Functional Materials (ICQD), University of Science and Technology of China, Hefei, 230026 Anhui China; 3 Department of Physics and Astronomy, Iowa State University, Ames, IA 50011 USA; 4 Fujian Provincial Key Laboratory of Theoretical and Computational Chemistry, Xiamen, China

**Keywords:** MoS_2_ multilayers, Electronic properties, Optical properties, Van Hove singularities

## Abstract

In this paper, the structural, electronic, and optical properties of MoS_2_ multilayers are investigated by employing the first-principles method. Up to six-layers of MoS_2_ have been comparatively studied. The covalency and ionicity in the MoS_2_ monolayer are shown to be stronger than those in the bulk. As the layer number is increased to two or above two, band splitting is significant due to the interlayer coupling. We found that long plateaus emerged in the imaginary parts of the dielectric function ${\varepsilon}_2^{xx}\left(\omega \right)$ and the joint density of states (JDOS) of MoS_2_ multilayers, due to the Van Hove singularities in a two-dimensional material. One, two and three small steps appear at the thresholds of both the long plateau of ${\varepsilon}_2^{xx}\left(\omega \right)$ and JDOS, for monolayer, bilayer, and trilayer, respectively. As the number of layers further increased, the number of small steps increases and the width of the small steps decreases accordingly. Due to interlayer coupling, the longest plateau and shortest plateau of JDOS are from the monolayer and bulk, respectively.

**Electronic supplementary material:**

The online version of this article (10.1186/s11671-019-3105-9) contains supplementary material, which is available to authorized users.

## Introduction

Molybdenum disulfide (MoS_2_) is one of the typical transition metal dichalcogenides and has been widely used as a catalyst [1] and hydrogen storage material [2, 3]. Owing to the strong in-plane interactions and weak van der Waals interactions between MoS_2_ atomic layers [4, 5], MoS_2_ crystals have been known as an important solid lubricant for many years [6, 7]. The monolayer MoS_2_, so-called 1*H*-MoS_2_, has been exfoliated from bulk MoS_2_ by using micromechanical cleavage [8]. The so-called 2*H*-MoS_2_ (among *1T*, *2H*, *3R*) is the most stable structure of bulk MoS_2_ [9, 10] and is a semiconductor with an indirect bandgap of 1.29 eV [4, 11, 12]. The monolayer MoS_2_ has also drawn great attention due to its two-dimensional nature and graphene-like honeycomb structure. It is interesting that monolayer MoS_2_ has a direct bandgap of 1.90 eV [4, 13] which can be used as a conductive channel of field-effect transistors [14]. On the other hand, the zero band gap of graphene restricts its applications in optics and transistor application [15–18]. Moreover, the theoretical and experimental works show that the electronic bandgap decreases as the number of MoS_2_ layers is increased [19–22]. Interlayer coupling of multilayer MoS_2_ is sensitive to layer thickness [21]. Some investigations on the multilayer MoS_2_ are available [19–25]; however, the electronic structures and optical properties of multilayer MoS_2_ are still not well-established, especially for the layer-dependent physical properties related to the interlayer coupling. Van Hove singularity (VHS) plays an important role in optical properties [26, 27] . The only available critical points in two-dimensional materials are those of the *P*_0_ (*P*_2_) and *P*_1_ type, which show as a step and a logarithmic singularity [26, 27]. In this paper, we analyze the electronic and optical properties of MoS_2_ related to Van Hove singularity, layer by layer and up to six atomic layers.

Nowadays, first-principles calculations have been successfully performed to study the structural, electronic, and optical properties of a wide variety of materials. In this work, we have systematically studied the electronic and optical properties of monolayer, multilayer and bulk MoS_2_ by using ab initio calculations. Discussions on the optical properties are emphasized. Our results show that, for **E**||*x*, the imaginary parts of the dielectric function ${\varepsilon}_2^{xx}\left(\omega \right)$ possess long plateaus. At these thresholds of these plateaus, ${\varepsilon}_2^{xx}\left(\omega \right)$ of the monolayer, bilayer, and trilayer exhibit one, two, and three small steps, respectively. The imaginary part of the dielectric function is also analyzed by the joint density of states and the transition matrix elements. JDOS combined with the band structures and the Van Hove singularities are discussed in detail.

## Methods

The present calculations have been performed by using the Vienna ab initio simulation package (VASP) [28, 29], which is based on the density functional theory, the plane-wave basis and the projector augmented wave (PAW) representation [30]. The exchange-correlation potential is treated within the generalized gradient approximation (GGA) in the form of Perdew-Burke-Ernzerhof (PBE) functional [31]. In order to consider the weak interlayer attractions in this layered crystal, PBE-D2 calculations [32] which include the semi-empirical van der Waals correction have been performed. In order to obtain more accurate band gaps, the Heyd-Scuseria-Ernzerhof hybrid functional (HSE06) [33–36] calculations are also performed in this work. The wave-functions of all the calculated systems are expanded in plane waves, with a kinetic energy cutoff of 500 eV. Brillouin zone (BZ) integrations are calculated by using a special **k**-point sampling of the Monkhorst-Pack scheme [37], with a 45 × 45 × 1 *Γ*-centered grid for the monolayer and multilayer MoS_2_ and 45 × 45 × 11 grid for the bulk MoS_2_ for PBE-D2 calculations. For HSE06 calculations, a 9 × 9 × 1 *Γ*-centered grid is used for the monolayer and multilayer MoS_2_. For the monolayer and multilayer MoS_2_, all the calculations are modeled by a supercell with a vacuum space of 35 Å in the *Z*-direction to avoid the interactions between adjacent MoS_2_ slabs. All the atomic configurations are fully relaxed until the Hellmann-Feynman forces on all the atoms are smaller than 0.01 eV/Å. Our spin-polarized calculations show that the band structures of MoS_2_ multilayers are rather insensitive to the spin-polarized effect (see Additional file 1: Figure S1); therefore, all the calculation results presented are based on the non-spin-polarization scheme.

**Fig. 1:**
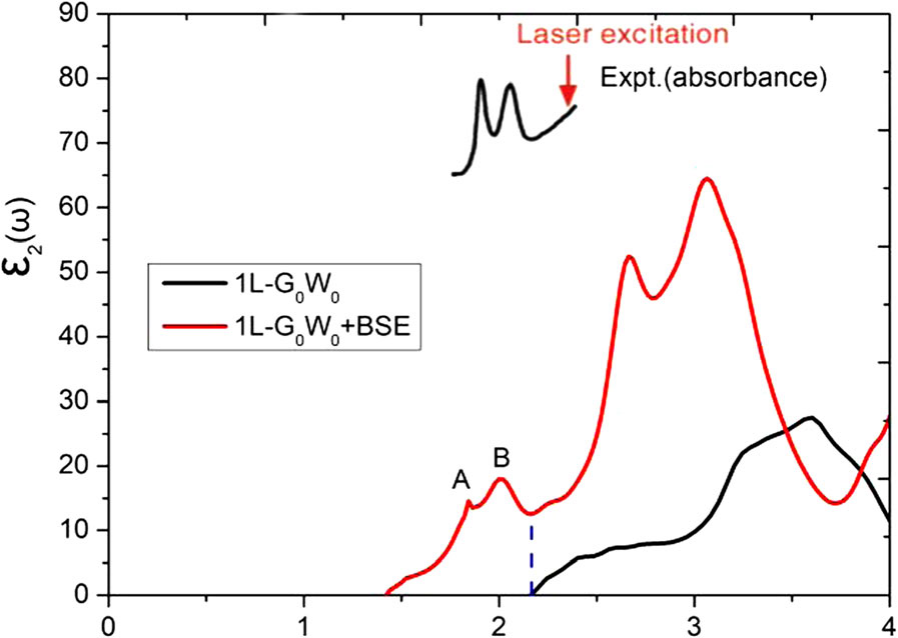
The imaginary parts of the dielectric function for monolayer MoS_2_, by using the G_0_W_0_ and G_0_W_0_+BSE methods, respectively. The experimental absorption spectrum for MoS_2_ is extracted from Ref. [4]

Excitonic effects in monolayer MoS_2_ are found to be significant and have been observed by photoluminescence. We have employed the quasi-particle G_0_W_0_ method [38], and the Bethe-Salpeter equation (BSE) [39, 40] to account for the excitonic effects. The band gaps of monolayer MoS_2_ are calculated to be 2.32 and 2.27 eV for the **k**-point meshes of 15 × 15 × 1 and 24 × 24 × 1 *Γ*-centered grid, obtained by the G_0_W_0_ with SOC calculations. The imaginary parts of the dielectric function are shown in Fig. 1, calculated from both the G_0_W_0_ and the G_0_W_0_ + BSE methods. Two exciton peaks at 1.84 and 1.99 eV are found, which agrees well with experimental observations [4, 41]. Although the G_0_W_0_+BSE scheme could describe the excitonic effects better, in this paper, we present only the results (without excitonic peaks) under the GGA-PBE functional.

## Results and Discussion

### Electronic Structures of MoS_2_ Multilayers

**Fig. 2:**
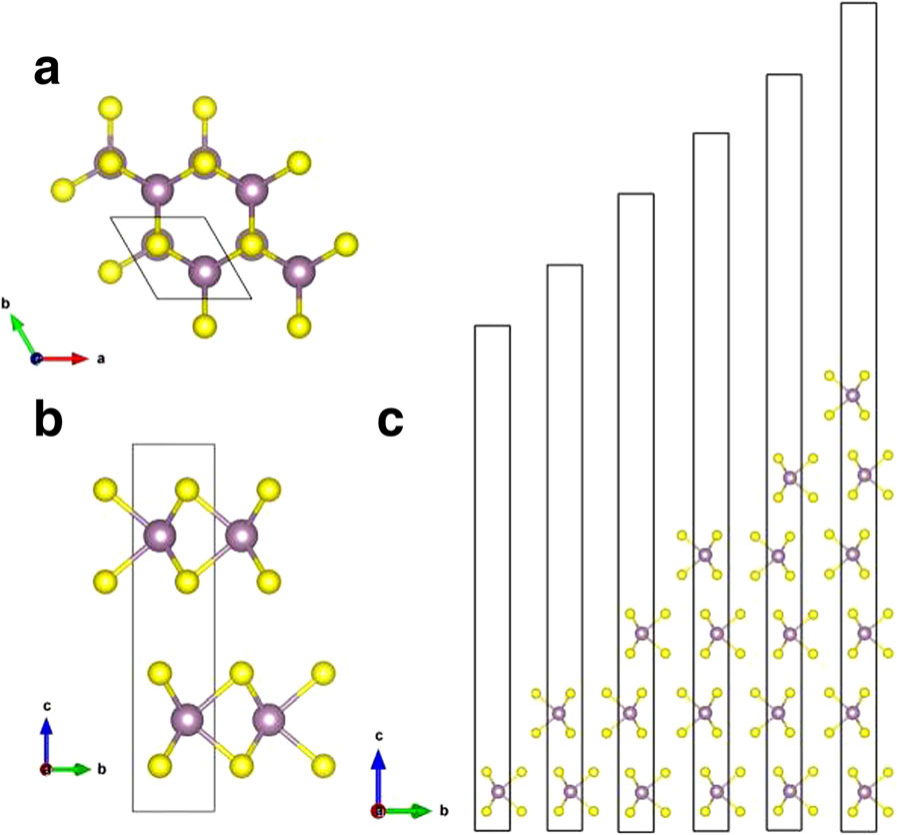
**a** Top view and **b** side view of bulk-MoS_2_. **c** Side view of monolayer, bilayer, trilayer, as well as four-, five-, and six-layered structures of MoS_2_. A unit cell is shown in **b**. Purple and yellow balls represent Mo and S atoms, respectively

Crystalline MoS_2_ occurs naturally and has three crystalline types: 1*T*, 2*H*, and 3*R*, which corresponds to crystals with trigonal, hexagonal, and rhombohedral primitive unit cells, respectively [9]. 2*H*-MoS_2_ is known as the most stable structure [10]; therefore, we consider only the 2*H* type of bulk MoS_2_ in this work. Bulk 2*H*-MoS_2_ has a hexagonal-layered structure consisting of layers of molybdenum atoms surrounded by six sulfur atoms, with S-Mo-S sheets piled up oppositely (showed in Fig. 2). The neighboring sheets in bulk 2*H*-MoS_2_ are weakly connected with weak van der Waals interactions. A monolayer MoS_2_ can then be easily exfoliated from the bulk. The lattice constants of bulk MoS_2_ are calculated to be *a = b* = 3.19Å, *c* = 12.41 Å, which are consistent with the reported values of *a = b* = 3.18 Å, *c* = 13.83 Å [18]. The optimized lattice constants for monolayer MoS_2_ are *a = b* = 3.19 Å, which are in accord with the bulk MoS_2_. As shown in Table 1, the calculated lattice constants in the *a*, *b* directions are the same for different number of layers of MoS_2_. It was also reported by Kumar et al. [19] that the lattice constants (*a, b*) of monolayer MoS_2_ are nearly identical to the bulk.

**Table 1:** Geometrical parameters, band gaps and the static dielectric constants for bulk and multilayer MoS_2_. Eg_1_, Eg_2_, Eg_3_, and Eg_4_ are the band gaps calculated by our PBE-D2 method, by our HSE06 method, by the GGA-PW91 method, and from the experimental data, respectively

	1L	2L	3L	4L	5L	6L	Bulk
*a* (Å)	3.19	3.19	3.19	3.19	3.19	3.19	3.19
*d*(Mo−S) (Å)	2.41	2.41	2.41	2.41	2.41	2.41	2.41
Eg_1_ (eV)	1.64	1.17	1.08	1.03	1.01	0.99	0.93
Eg_2_ (eV)	2.09	1.66	1.56	1.50	1.49	1.48	1.45
Eg_3_ (eV)	1.57[22]	1.20[22]	–	–	–	–	0.89[22]
Eg_4_ (eV)	1.90[4], 1.82[20]	1.60[4], 1.65[20]	1.46[4], 1.35[20]	1.41[4]	1.37[4]	1.35[4]	1.29[4], 1.23[11]
${\varepsilon}_1^{xx}(0)$	15.29	15.44	15.45	15.48	15.48	15.47	15.57

**Fig. 3:**
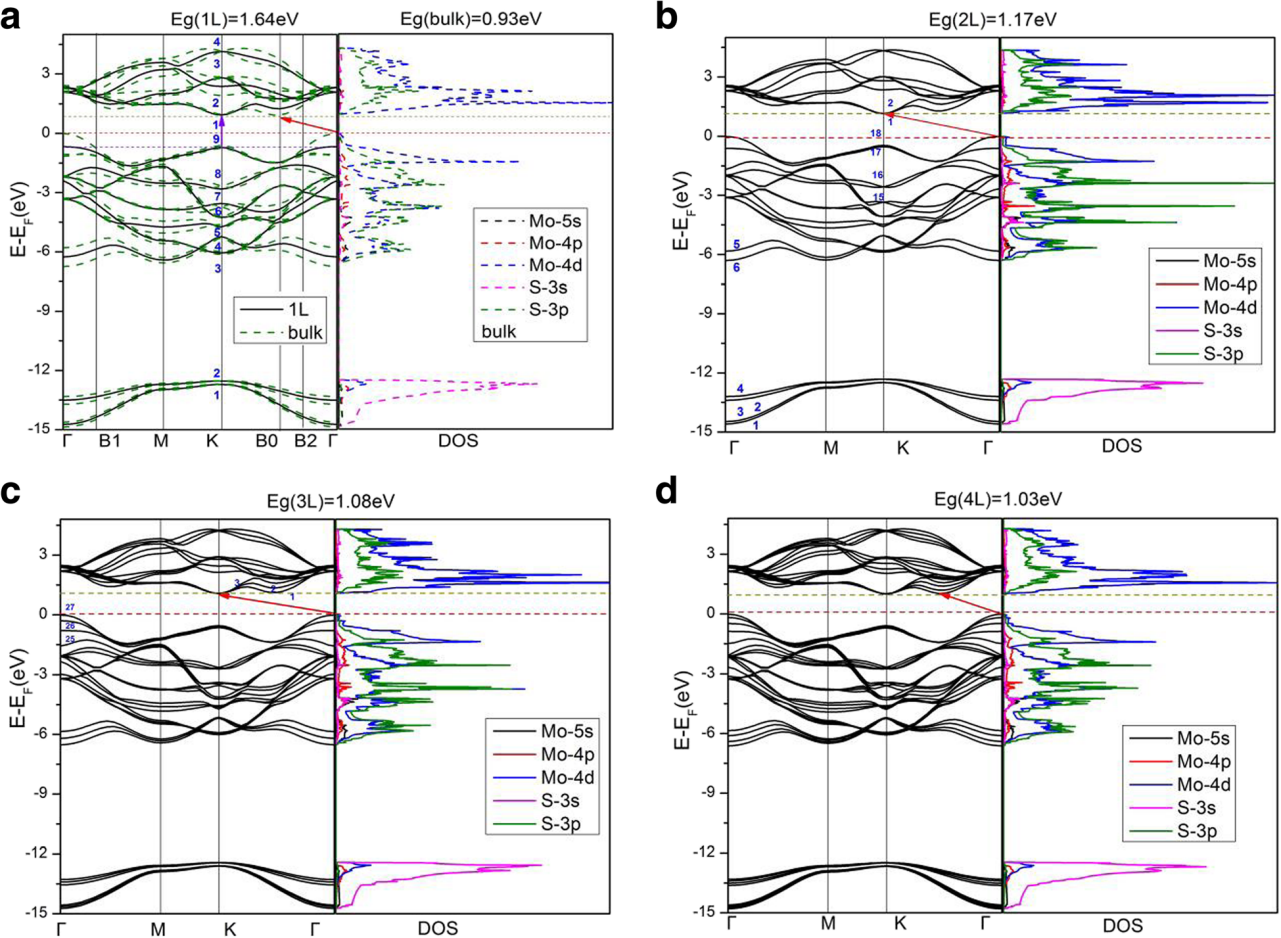
Calculated band structures and density of states of **a** monolayer (full lines) and bulk (dash lines), **b** bilayer, **c** trilayer, and **d** four-layer MoS_2_. In **a**, the highest occupied bands for bulk and monolayer at point K are set to the same energy. Conduction band minimum of bulk is at point B0

**Fig. 4:**
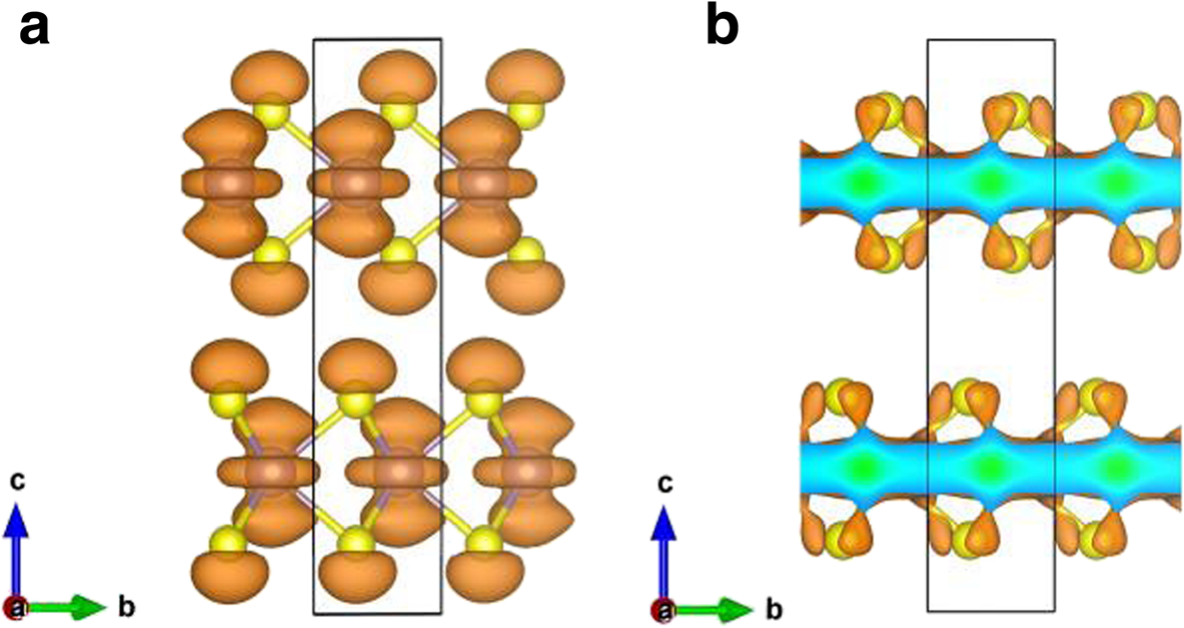
The charge distributions of the highest occupied band at **a** point  and **b** point K for bulk MoS_2_. The isosurface value is set to be 0.004 e/Å^3^

Figure 3 depicts the calculated band structures and electronic density of states (DOS) of different number of layers of MoS_2_. Results for monolayer, bilayer, trilayer, and four-layer as well as bulk MoS_2_ are given in Fig. 3, while results for five-layer and six-layer MoS_2_ are very similar to those of four-layer and bulk. For monolayer MoS_2_, both the valence band maximum (VBM) and the conduction band minimum (CBM) appear at K-point of the BZ, exhibiting a direct bandgap of 1.64 eV. For bilayer and trilayer MoS_2_, both the VBM locates at Γ point while both the CBM lies at K point, causing indirect gaps of 1.17 and 1.08 eV, respectively. However, as the number of MoS_2_ layers increases to four and above four, all the multilayers MoS_2_ show same characters that the VBM locates at Γ point while the CBM lies between Γ and K points, which is the same as in the bulk. Indirect band gaps are 1.03 eV, 1.01 eV, 0.99 eV, 0.93 eV for four-, five-, six-layer MoS_2_, and bulk, respectively. Both the PBE-D2 and HSE06 calculations (Table 1) show that the fundamental band gap increases monotonically when the number of MoS_2_ layers decreases, which is due to a large confinement of electrons in the slab [4, 5, 19, 42]. Moreover, when the bulk MoS_2_ slab is lessened to a single-layer, it turns into a direct bandgap semiconductor, as mentioned previously, the bulk MoS_2_ is an indirect gap semiconductor. In Fig. 3a, band structures plot of bulk MoS_2_ show splitting of bands (as compared to those of monolayer MoS_2_), mainly around the -point, owing to interlayer coupling [16]. Band structures for two-layers (2L) and more than 2L MoS_2_ exhibit similar splitting of bands owing again to the interlayer coupling. However, splitting of bands in the bulk is somewhat more significant than those in the multilayers MoS_2_, indicating a (slightly) stronger interlayer coupling in the bulk than in the multilayers. On the other hand, splitting of bands in the vicinity of point K in BZ is very small. The electronic states at point K for the highest occupied band are mainly composed of *d*_*xy*_ and ${d}_{x^2-{y}^2}$ orbitals of Mo atoms, as well as small parts of (*p*_*x*_, *p*_*y*_)-orbitals of S atoms (shown in Fig. 4b). The Mo atoms are situated in the middle layer of S-Mo-S sheet, which causes a negligible interlayer coupling at K point (since the nearest atoms between MoS_2_ layers are S and S). As shown in Fig. 4, stronger interlayer coupling at point Γ can be found when compared with that at point K, since electronic states at point Γ for the highest occupied band are dominated by ${d}_{z^2}$ orbitals of Mo atoms and *p*_*z*_ orbitals of S atoms. Therefore, S-S coupling (interlayer coupling) is clearly stronger at point Γ than that at point K. Our results are consistent with other theoretical work [21]. Generally speaking, the electronic density of states of few-layer MoS_2_ are similar to those of bulk MoS_2_ (see Fig. 3), since bulk MoS_2_ is actually a layered material with weak interactions between the MoS_2_ layers.

**Fig. 5:**
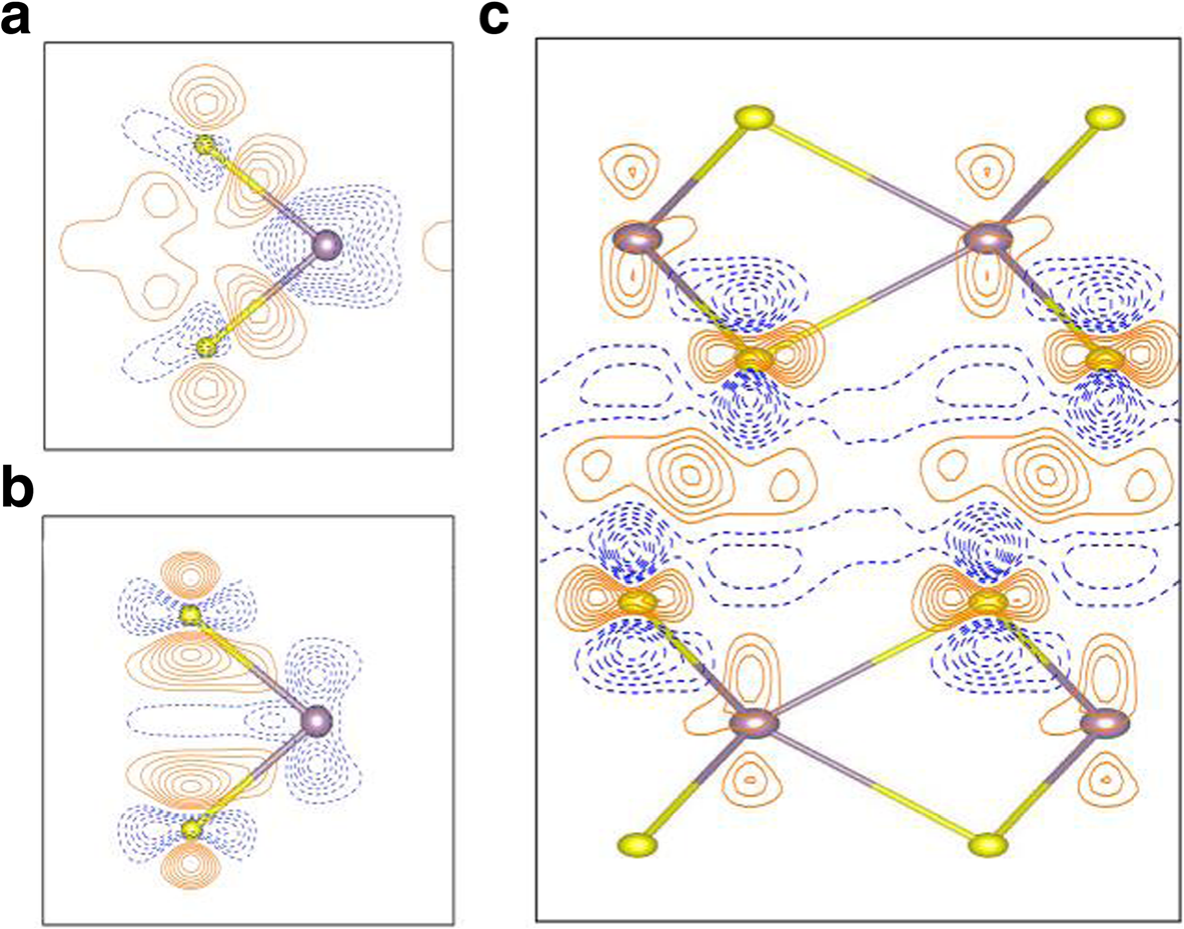
**a** Deformation charge density, Δ*ρ*_1_(**r**) = *ρ*(**r**) − ∑_*μ*_*ρ*_atom_(**r** − **R**_*μ*_), in the monolayer MoS_2_. **b** Differences between the charge densities of monolayer and the corresponding layer of bulk. **c** The interlayer charge density redistribution of bilayer MoS_2_. Contour interval of **a** is 2.5 × 10^−2^ e/Å^3^, while both those of **b** and **c** are 2.5 × 10^−4^ e/Å^3^. Solid orange and dashed blue lines correspond to Δ*ρ* > 0 and Δ*ρ* < 0_,_ respectively

To deeply explore the bonding nature in the monolayer MoS_2_, the deformation charge density is shown in Fig. 5a. The deformation charge density is given by *Δρ*_1_(**r**) = *ρ*(**r**) − ∑_*μ*_*ρ*_atom_(**r** − **R**_*μ*_) where *ρ*(**r**) is the total charge density and ∑_*μ*_*ρ*_atom_(**r** − **R**_*u*_) stands for the superposition of independent atomic charge densities. The results demonstrate that the bonding in the MoS_2_ monolayer is characterized by clear covalent (solid contours lines in between the Mo-S atoms), as well as strong ionic interactions (represented by alternating areas of dashed and solid contours). To see the bonding strength in the monolayer MoS_2_ as compared to those in the bulk, the charge density differences between monolayer and bulk MoS_2_, *Δρ*_2_(**r**), is also presented in Fig. 5b. The charge density difference is defined as *Δρ*_2_(**r**) = *ρ*_1*L*_(**r**) − *ρ*_bulk_(**r**), where *ρ*_1*L*_(**r**) and *ρ*_bulk_(**r**) are the total charge densities of monolayer and bulk MoS_2_, respectively. Figure 5b indicates a stronger electronic binding in the monolayer case than those in the bulk, which is reflected by the larger charge accumulation (solid contours lines) in between the Mo-S atoms in the monolayer, as well as by stronger ionic bonding in the monolayer MoS_2_ since the alternating areas of dashed and solid contours in the Fig. 5b are more significant than those in the bulk. Moreover, the charge differences plot (Fig. 5b) indicates that Mo atom of monolayer lost more electrons than Mo atom in the bulk; therefore, the ionicity of monolayer is stronger than bulk. However, it should be pointed out that the order of magnitude of the charge differences in the Fig. 5b are fairly small (the contour interval in the Fig. 5b is only 2.5 × 10^−4^ e/Å^3^). Judge from the quantum confinement effect, again, the intra-layer interaction of monolayer should be stronger than bulk. Hence, the bandgap of the monolayer (1.64 eV) is expected to be larger than bulk (0.93 eV). Quantum confinement decreases with the increasing layer number [4, 42], which enhances interlayer coupling and reduce intra-layer interaction. Thus, the band gap of MoS_2_ decreases with the increase of interlayer coupling. The interlayer charge density redistributions for bilayer MoS_2_, *Δρ*_3_(**r**), are also presented in Fig. 5c. The *Δρ*_3_(**r**) is given by *Δρ*_3_(**r**) = *ρ*_2*L*_(**r**) − *ρ*_layer1_(**r**) − *ρ*_layer2_(**r**), where *ρ*_2*L*_(**r**), *ρ*_layer1_(**r**), *ρ*_layer2_(**r**) are the charge densities of the bilayer MoS_2_, the first layer of bilayer MoS_2_ and the second layer of bilayer MoS_2_, respectively. The charge densities of layer1 and layer2 of bilayer MoS_2_ are calculated by using the corresponding structure in bilayer MoS_2_. Charge transfer from MoS_2_ layers (bilayer) to the intermediate region between the MoS_2_ layers are clearly seen in Fig. 5c, shown as solid contour lines. The ionic interactions between atomic layers in bilayer MoS_2_ are also clear, as seen from the alternating areas of dashed and solid contours. Again, the order of magnitude of interlayer charge densities, *Δρ*_3_(**r**), are very small (the contour interval is only 2.5 × 10^-4^ e/Å^3^). Generally, the inter-layer charge density redistributions in 2L, 3L, …, bulk MoS_2_ systems are all very similar.

### Optical Properties of MoS_2_ Multilayers

Once the ground state electronic structures of a material are obtained, the optical properties can then be investigated. The imaginary part of the dielectric function ${\varepsilon}_2^{\alpha \beta}\left(\omega \right)$ is determined by the following equation [43]:

1 $$ {\displaystyle \begin{array}{c}{\varepsilon}_2^{\alpha \beta}\left(\omega \right)=\frac{4{\pi}^2{e}^2}{\Omega}{\lim}_{q\to 0}\frac{1}{q^2}\underset{c,v,k}{\Sigma}2{w}_k\delta \left({E}_{ck}-{E}_{vk}-\mathrm{\hslash}\omega \right)\\ {}\times \left\langle {u}_{ck+{e}_{\alpha }q}|{u}_{vk}\right\rangle \left\langle {u}_{ck+{e}_{\beta }q}|{u}_{vk}\right\rangle \ast \end{array}} $$

where the indices *α* and *β* denote Cartesian directions, *c* and *v* refer to conduction and valence bands, *E*_*ck*_ and *E*_*vk*_ are the energies of conduction bands and valence bands, respectively. The Kramers-Kronig inversion can be applied to acquire the real part of the dielectric function ${\varepsilon}_1^{\alpha \beta}\left(\omega \right)$ determined by the imaginary part ${\varepsilon}_2^{\alpha \beta}\left(\omega \right)$:

2 $$ {\varepsilon}_1^{\alpha \beta}\left(\omega \right)=1+\frac{2}{\pi }P{\int}_0^{\infty}\frac{\varepsilon_2^{\alpha \beta}\left(\omega \hbox{'}\right)\omega \hbox{'}}{\omega {\hbox{'}}^2-{\omega}^2+ i\eta} d\omega \hbox{'} $$

in which P represents the principal value. Since MoS_2_ has a uniaxial structure, *ε*^*xx*^(*ω*) is then identical to *ε*^*yy*^(*ω*). In this work, we need only discuss the electric vector **E **which is parallel the *x-y* plane, i.e., **E||**x is parallel to the MoS_2_ *x-y* plane.

For investigating detailed optical spectra of MoS_2_ system, the absorption coefficient *α*(*ω*) and the reflectivity *R*(*ω*) were calculated by the real part *ε*_1_(*ω*) and the imaginary part  of the dielectric function. Equations of parameters mentioned are presented below:

3 $$ \alpha \left(\omega \right)=\sqrt{2}\frac{\omega }{c}\sqrt{\sqrt{\varepsilon_1^2\left(\omega \right)+{\varepsilon}_2^2\left(\omega \right)}-{\varepsilon}_1\left(\omega \right)} $$

4 $$ R\left(\omega \right)={\left|\frac{\sqrt{\varepsilon_1\left(\omega \right)+i{\varepsilon}_2\left(\omega \right)}-1}{\sqrt{\varepsilon_1\left(\omega \right)+i{\varepsilon}_2\left(\omega \right)}+1}\right|}^2 $$

If the matrix element $\left\langle {u}_{ck+{e}_{\alpha }q}|{u}_{vk}\right\rangle$ varies very slowly as **k**-vector, the term $\left\langle {u}_{ck+{e}_{\alpha }q}|{u}_{vk}\right\rangle \left\langle {u}_{ck+{e}_{\beta }q}|{u}_{vk}\right\rangle \ast$ in Eq. (1) can be taken outside the summation. In Eq. (1), most of the dispersion in ${\varepsilon}_2^{\alpha \beta}\left(\omega \right)$ is due to the summation over the delta function *δ*(*E*_*ck*_ − *E*_*vk*_ − ℏ*ω*). This summation can be transformed into an integration over energy by defining a joint density of states (JDOS) [25, 44],

5 $$ {J}_{cv}\left(\omega \right)=\frac{1}{4{\pi}^3}\int \frac{dS_k}{\nabla_k\left({E}_{ck}-{E}_{vk}\right)} $$

in which ℏ*ω* equals *E*_*ck*_ − *E*_*vk*_, *S*_*k*_ represents the constant energy surface denoted by *E*_*ck*_ − *E*_*vk*_ = ℏ*ω* = const. The joint density of states *J*_*cv*_(*ω*) is associated with the transitions from the valence bands to the conduction bands, and the large peaks in *J*_*cv*_(*ω*) will originate in the spectrum where ∇_*k*_(*E*_*ck*_ − *E*_*vk*_) ≈ 0. Points in **k**-space where ∇_*k*_(*E*_*ck*_ − *E*_*vk*_) = 0 are called critical points or van Hove singularities (VHS), and *E*_*ck*_ − *E*_*vk*_ are called critical point energies [26, 27]. The critical points ∇_*k*_*E*_*ck*_ = ∇_*k*_*E*_*vk*_ = 0 usually occur only at high-symmetry points, while critical points ∇_*k*_*E*_*ck*_ = ∇_*k*_*E*_*vk*_ ≠ 0 may occur at any general points in the Brillouin zone [27, 45]. In the two-dimensional case, there are three types of critical points, i.e., *P*_0_ (minimum point), *P*_1_ (saddle point), and *P*_2_ (maximum point). At the points *P*_0_ or *P*_2_, a step function singularity occurred in JDOS, while at the saddle point *P*_1_, JDOS was described by a logarithmic singularity [27]. In more detail, the *E*_*c*_(*k*_*x*_, *k*_*y*_) − *E*_*v*_(*k*_*x*_, *k*_*y*_) can be expanded in a Taylor series about the critical point. Limiting the expansion to quadratic terms, with the linear term does not occur due to property of the singularity, we then have

6 $$ {E}_c\left({k}_x,{k}_y\right)-{E}_v\left({k}_x,{k}_y\right)={E}_0+\frac{\mathrm{\hslash}}{2}\left({b}_x\frac{k_x^2}{m_x}+{b}_y\frac{k_y^2}{m_y}\right) $$

Therefore, three types of critical points emerge. For *P*_0_, (*b*_*x*_ > 0, *b*_*y*_ > 0), for *P*_1_, (*b*_*x*_ > 0, *b*_*y*_ < 0) or (*b*_*x*_ < 0, *b*_*y*_ > 0), and for *P*_2_, (*b*_*x*_ < 0, *b*_*y*_ < 0). In this paper, for the case of MoS_2_ multilayers, only the *P*_0_ critical point is involved.

**Fig. 6:**
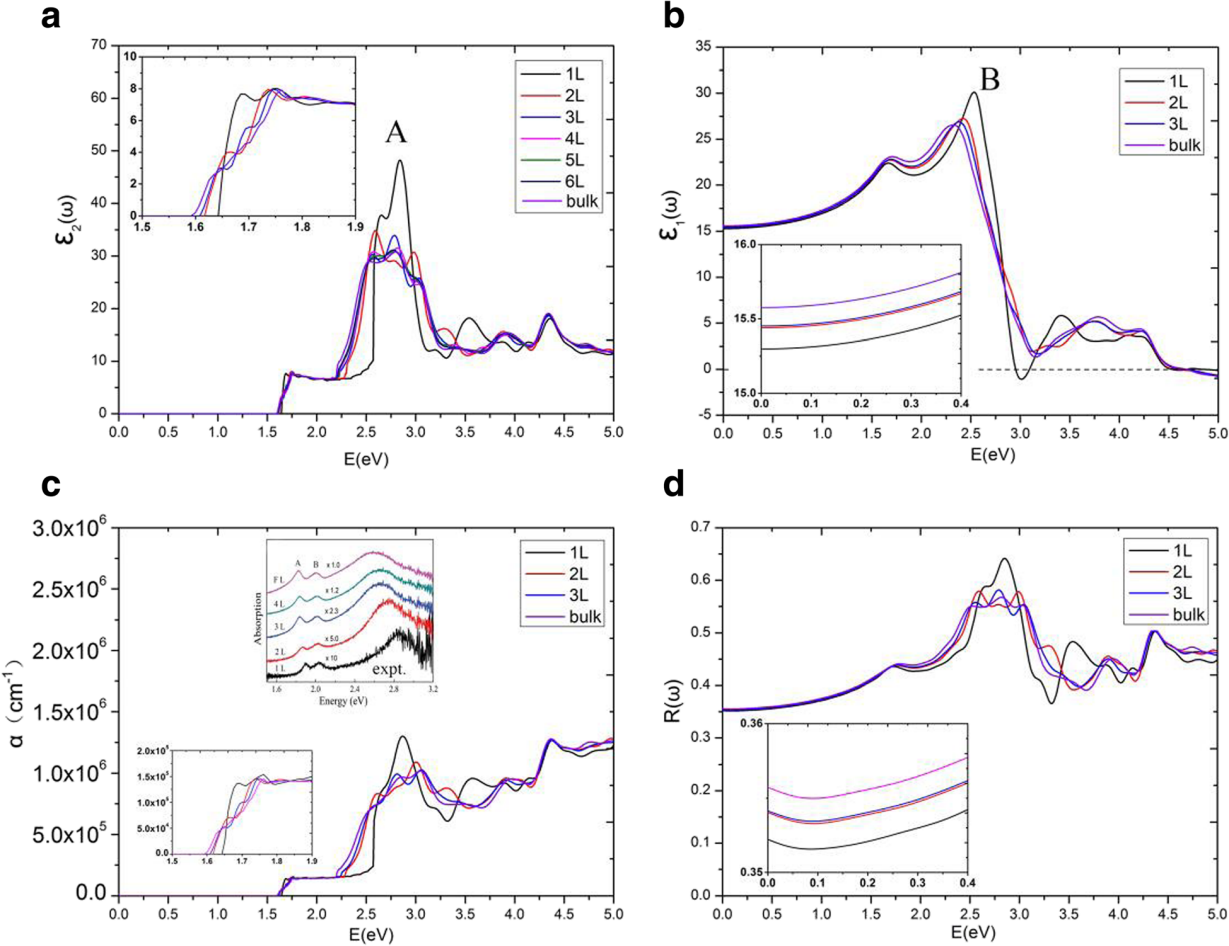
**a** The imaginary parts of the dielectric function, **b** the real parts of the dielectric function, **c** the absorption coefficients, and **d** the reflectivity spectra, for different number of MoS_2_ layers. The inset in **c** also shows the experimental data [46]

**Fig. 7:**
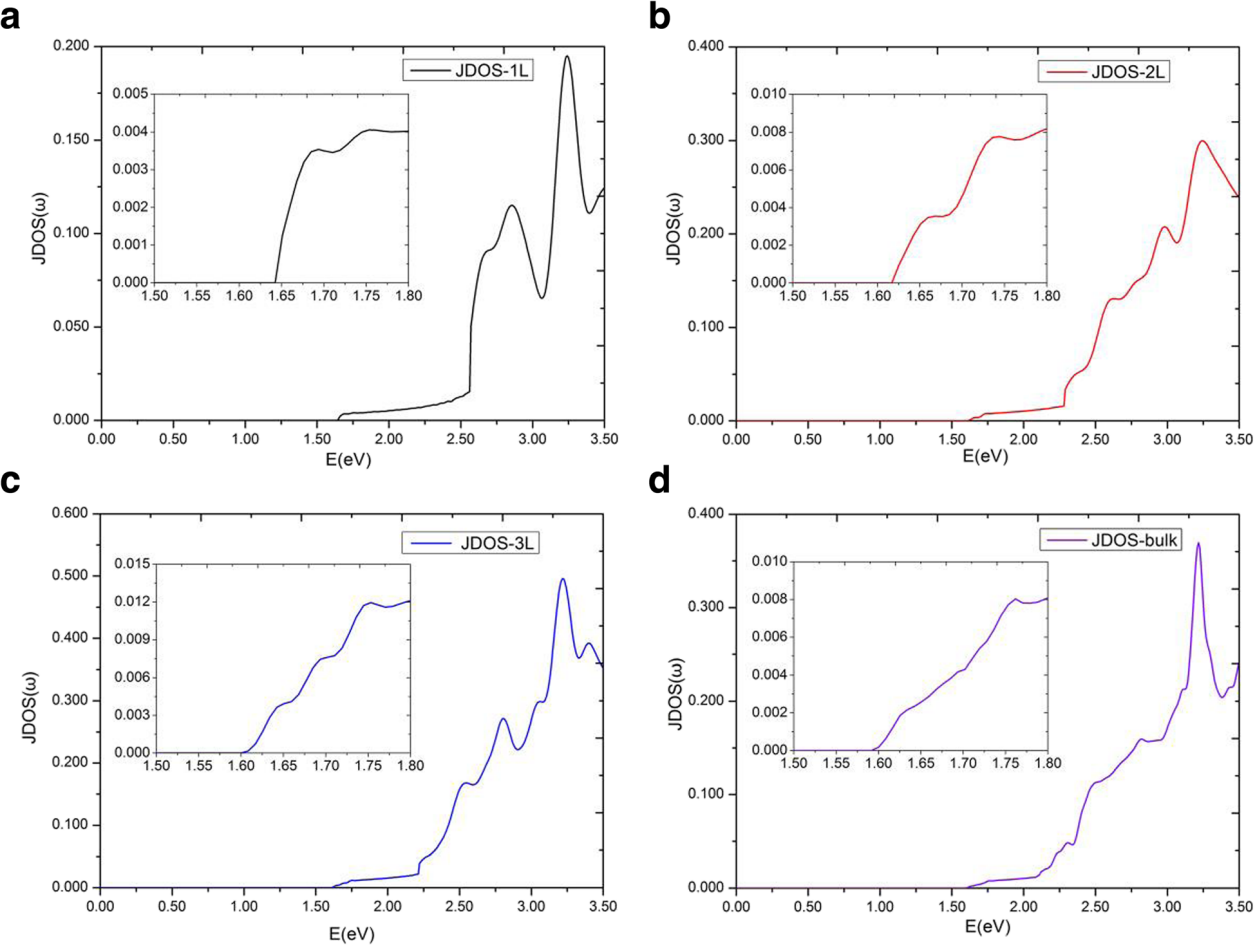
Joint density of states for the monolayer, bilayer, trilayer, and bulk MoS_2_

Figure 6a gives the imaginary parts of dielectric function, ${\varepsilon}_2^{xx}\left(\omega \right)$, of MoS_2_ multilayers for **E**||x. We found an interesting phenomenon that the imaginary parts of dielectric function ${\varepsilon}_2^{xx}\left(\omega \right)$ possess plateaus, and the plateaus of different layers of MoS_2_ are nearly equal in the range of 1.75 eV~2.19 eV. From the threshold energy up to 1.75 eV, ${\varepsilon}_2^{xx}\left(\omega \right)$ are quite different for different multilayers of MoS_2_. The threshold and ending energies of the plateaus in different layers are different, especially, the energy range of ${\varepsilon}_2^{xx}\left(\omega \right)$ plateau of the monolayer is significantly broader than those of other multilayers. The threshold energy of monolayer MoS_2_ dielectric function is equal to its direct bandgap of 1.64 eV. However, the threshold energy of bilayer dielectric function is not the indirect bandgap of 1.17 eV but the minimum of direct energy gap of 1.62 eV between the valence and conduction bands. This is because that we study only the transitions between valence and conduction bands with the same electron wave vector, which are classified as direct optical transitions [36, 47]. As the number of MoS_2_ layers increased to 4, we found that ${\varepsilon}_2^{xx}\left(\omega \right)$ of multilayer MoS_2_ systems were almost indistinguishable from bulk. Hence, we discuss here in details only the plateaus of the monolayer, bilayer, and trilayer, as well as bulk MoS_2_. The ${\varepsilon}_2^{xx}\left(\omega \right)$ plateaus of monolayer, bilayer, trilayer, and bulk MoS_2_ ended at 2.57 eV, 2.28 eV, 2.21 eV, and 2.19 eV, respectively. To explain this more precisely, JDOS of monolayer, bilayer, trilayer, and bulk MoS_2_ are shown in Fig. 7. From Fig. 7, the plateaus are also shown to be in the JDOS. The plateaus of monolayer, bilayer, and trilayer JDOS ended at 2.57 eV, 2.28 eV, 2.21 eV, respectively, which are exactly the same as those in their ${\varepsilon}_2^{xx}\left(\omega \right)$. For bulk MoS_2_, the plateau of JDOS ended at 2.09 eV, which is slightly smaller than 2.19 eV in the dielectric function ${\varepsilon}_2^{xx}\left(\omega \right)$.

**Fig. 8:**
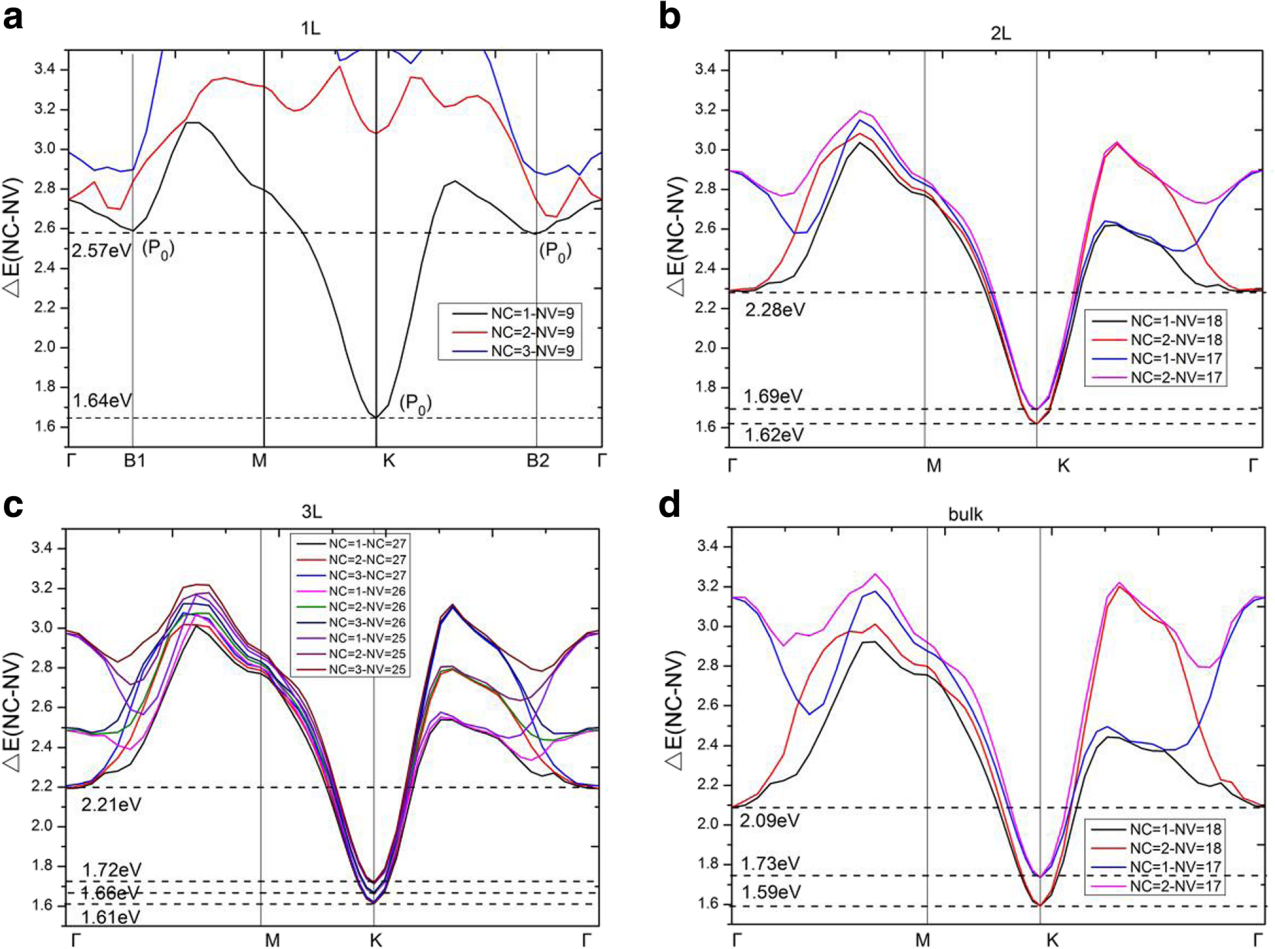
Direct energy gaps, ΔE(NC − NV), between conduction and valence bands for the **a** monolayer, **b** bilayer, **c** trilayer, and **d** bulk MoS_2_. **a**–**d** There are three, six, twelve, and six critical points in interband transitions for the monolayer, bilayer, trilayer, and bulk MoS_2_, respectively

To analyze accurately the electronic transitions and for a detailed analysis of the dielectric function ${\varepsilon}_2^{xx}\left(\omega \right)$, the direct energy gaps, ΔE(NC − NV), between conduction and valence bands of monolayer, bilayer, trilayer, and bulk MoS_2_ are presented in Fig. 8. The notations NC and NV represent the ordinal numbers of conduction and valence bands. Hence, NC = 1, 2, and 3 signify the lowest, the second lowest, and the third lowest unoccupied band of material. On the other hand, NV = 9, 18, and 27 (which is dependent on the number of electrons in the unit cell) signify the highest occupied band of monolayer, bilayer, and trilayer MoS_2_, respectively. For monolayer, in the region of 0 ~ 2.57 eV, the electronic transitions are found to be contributed only from the highest occupied band NV = 9 to the lowest unoccupied band NC = 1. From Fig. 8a, a minimum appears at high symmetry point K and the threshold of JDOS (Fig. 7a) appears at 1.64 eV which is actually the direct bandgap of the monolayer MoS_2_. In the vicinity of high symmetry point K, the curve of ΔE(NC = 1 − NV = 9) is similar to a parabola for monolayer MoS_2_. Therefore, ∇_*k*_(*E*_*ck*_ − *E*_*vk*_) = 0 at K point, which means a critical point at high symmetry point K. In a two-dimensional structure, this critical point belongs to *P*_0_ type singularity [27], and therefore it leads to a step in the JDOS. Thus, the threshold energy of the JDOS plateau is found at critical point energy 1.64 eV. The ending energy of the JDOS plateau is near 2.57 eV, which is resulted from the appearance of two *P*_0_ type singularities at point B1 (**k** = (0.00, 0.16, 0.00)) and point B2 (**k** = (− 0.10, 0.20, 0.00)). The slopes of the ΔE(NC = 1 − NV = 9) curve near the two critical points B1 and B2 are very small, which give rise to a rapid increase in JDOS (see Eq.(5)). Main critical points for these long plateaus of JDOS are listed in Table 2, including type, location, transition bands, and the direct energy gap ΔE(NC − NV). Furthermore, we found that ∇_*k*_*E*_*ck*_ = ∇_*k*_*E*_*vk*_ = 0 happened at high symmetry point K where the slopes of the valence and conduction bands are horizontal. While ∇_*k*_*E*_*ck*_ = ∇_*k*_*E*_*vk*_ ≠ 0 happened at points B1 and B2, which means that slopes of two bands are parallel. Simultaneously, analysis on the band structures and direct energy gaps (see Fig. 8a) for the monolayer show that, when the direct energy gap ΔE is below 2.65 eV, only the transitions between NV = 9 and NC = 1 contribute to JDOS; when the ΔE is larger than 2.65 eV, the transitions of NV = 9 to NC = 2 also begin to contribute to JDOS; while when the ΔE reaches above 2.86 eV, the NV = 9 to NC = 3 transitions have effect on JDOS. It should be pointed out that for energy larger than 2.65 eV, many bands in Fig. 8a will contribute to JDOS. JDOS of monolayer MoS_2_ exhibits a plateau in the range of 1.64 ~ 2.57 eV and the variation of the expression |M_vc_|^2^/*ω*^2^ is found to be small in this range. According to Eqs. (1) and (5), the imaginary part of the dielectric function ${\varepsilon}_2^{xx}\left(\omega \right)$ is mainly decided by the JDOS and the transition matrix elements, this gives a similar plateau for the imaginary part of dielectric function ${\varepsilon}_2^{xx}\left(\omega \right)$ as compared to JDOS.

**Table 2:** Main critical points in interband transitions below 3 eV. For the meaning of the parameters in the table, please refer to Fig. 3 and Fig. 8

	Type	Location	Interband transition	Direct energy gap △*E*(NC-NV)
1L	*P*_0_	K (− 0.33, 0.66, 0.00)	NV = 9→NC = 1	1.64 eV
	*P*_0_ *P*_0_	B1(Γ − M)(0.00, 0.16, 0.00) B2(Γ − K)(− 0.10, 0.20, 0.00)	NV = 9→NC = 1 NV =9→NC = 1	2.57 eV 2.57 eV
2L	*P*_0_ *P*_0_	K(− 0.33, 0.66, 0.00) K(− 0.33, 0.66, 0.00)	NV = 18→NC = 1, 2 NV = 17→NC=1, 2	1.62 eV 1.69 eV
	*P*_0_	Γ(0.00, 0.00, 0.00)	NV = 18→NC=1, 2	2.28 eV
3L	*P*_0_ *P*_0_ *P*_0_	K(− 0.33, 0.66, 0.00) K(− 0.33, 0.66, 0.00) K(− 0.33, 0.66, 0.00)	NV = 27→NC = 1, 2, 3 NV = 26→NC = 1, 2, 3 NV = 25→NC = 1, 2, 3	1.61 eV 1.66 eV 1.72 eV
	*P*_0_	Γ(0.00, 0.00, 0.00)	NV = 27→NC = 1, 2, 3	2.21 eV
Bulk	*P*_0_ *P*_0_	K(− 0.33, 0.66, 0.00) K(− 0.33, 0.66, 0.00)	NV = 18→NC = 1, 2 NV = 17→NC = 1, 2	1.59 eV 1.73 eV
	*P*_0_	Γ(0.00, 0.00, 0.00)	NV = 18→NC = 1, 2	2.09 eV

For bilayer MoS_2_, in the region of 0 ~ 2.28 eV (the endpoint of JDOS plateau), the electronic transitions are contributed to NV = 17, 18 to NC = 1, 2. The minimum energy in ΔE(NC − NV) is situated at the K point with a gap of 1.62 eV. In Fig. 8b, similar to monolayer MoS_2_, bilayer MoS_2_ holds two parabolic curves going upward (which come from ΔE(NC = 1 − NV = 18) and ΔE(NC = 2 − NV = 18)) at K point. Therefore, there are two *P*_0_ type singularities (∇_*k*_(*E*_*ck*_ − *E*_*vk*_) = 0) at K point, causing a step in the JDOS. The critical point energies are both 1.62 eV, this is because that the conduction bands (NC = 1 and NC = 2) are degenerate at point K (as shown in Fig. 3b), which results in the same direct energy gap between transitions of NV = 18 to NC = 1 and NV = 18 to NC = 2. From Fig. 8b, as the direct energy gap is increased to 1.69 eV, two new parabolas (which come from ΔE(NC = 1 − NV = 17) and ΔE(NC = 2 − NV = 17)) appear and two new singularities emerge again at K point in the direct energy gap graph, leading to a new step in JDOS for bilayer MoS_2_ (see Fig. 7b). As a result, the JDOS of the bilayer MoS_2_ has two steps around the threshold of long plateau (see inset in Fig. 7b). Two parabolas (in Fig. 8b) contribute to the first step and four parabolas contribute to the second step in JDOS. It means that the value of the second step is roughly the double of the first one. As the ΔE reaches to 2.28eV, two new singularities appear at Γ point (where interband transitions come from Γ(NV = 18→NC = 1) and Γ(NV = 18→NC = 2)), which have great contribution to the JDOS and bring the end to the plateau. Our calculations demonstrate that ∇_*k*_*E*_*ck*_ = ∇_*k*_*E*_*vk*_ = 0 are satisfied not only at high symmetry point K, but also at high symmetry point Γ. Similar to the case of monolayer, we found that the term of |M_vc_|^2^/*ω*^2^ is a slowly varying function in the energy range of bilayer JDOS plateau; hence, ${\varepsilon}_2^{xx}\left(\omega \right)$ of bilayer have a similar plateau in the energy range.

For trilayer MoS_2_, in the region of 0 ~ 2.21 eV, the JDOS are contributed from electronic transitions of NV = 25, 26, and 27 to NC = 1, 2, and 3. As shown in Fig. 8c, trilayer MoS_2_ have nine singularities at three different energies (ΔE = 1.61 eV, 1.66 eV, and 1.72 eV, respectively) at the K point. Figure 3c depicts that the conduction bands (NC = 1, 2, 3) are three-hold degenerate at point K; this means that there are three singularities at each critical point energy. According to our previous analysis, the JDOS and ${\varepsilon}_2^{xx}\left(\omega \right)$ of trilayer MoS_2_ will show three steps near the thresholds of the long plateaus, the endpoints of the long plateaus of trilayer JDOS, and ${\varepsilon}_2^{xx}\left(\omega \right)$ are then owing to the appearance of three singularities at Γ point with ΔE = 2.21 eV (see Fig. 7c), which come from the interband transitions of Γ(NV = 27→NC = 1, 2, 3).

For bulk MoS_2_, the thresholds of ${\varepsilon}_2^{xx}\left(\omega \right)$ and JDOS are also located at K point, with the smallest ΔE(NC − NV) equals to 1.59 eV. Nevertheless, there is no obvious step appeared in the thresholds of plateaus for both the ${\varepsilon}_2^{xx}\left(\omega \right)$ and JDOS (see Fig. 6a and Fig. 7d). Based on the previous analysis, the number of steps in the monolayer, bilayer, and trilayer MoS_2_ are 1, 2, and 3, respectively. As the number of MoS_2_ layers increases, the number of steps also increases in the vicinity of the threshold energy. Thus, in the bulk MoS_2_, the JDOS curve is composed of numerous small steps around the threshold energy of the long plateau, and finally the small steps disappear near the threshold energy since the width of the small steps decreases. In the region of 0 ~ 2.09 eV, the electron transitions of bulk MoS_2_ are contributed to NV = 17, 18 to NC = 1, 2. The 2.09 eV is the endpoint of JDOS plateau of bulk MoS_2_, which is attributed to two singularities, i.e., the interband transitions of Γ(NV = 18→NC = 1) as well as Γ(NV = 18→NC = 2), as presented in Fig. 8d. However, the plateau endpoint of the imaginary part of dielectric function ${\varepsilon}_2^{xx}\left(\omega \right)$ is 2.19 eV, which is greater than the counterpart of JDOS (e.g., 2.09 eV). Checked the transition matrix elements, it verified that some transitions are forbidden by the selection rule in the range of 2.09 eV to 2.19 eV. Therefore, the imaginary part of the dielectric function ${\varepsilon}_2^{xx}\left(\omega \right)$ is nearly invariable in the range of 2.09 ~ 2.19 eV. As a result, the plateau of ${\varepsilon}_2^{xx}\left(\omega \right)$ of bulk MoS_2_ is then 1.59 ~ 2.19 eV.

It has been shown that these thresholds of the JDOS plateaus are determined by singularities at the K point for all of the studied materials, see Fig. 8. The endpoint energy of the monolayer JDOS plateau is determined by two critical points at B1 and B2 (Fig. 8a). Nevertheless, the endpoint energies of bilayer, trilayer, and bulk JDOS plateaus are all dependent on the critical points at Γ(Fig. 8b–d). The interlayer coupling near point Γ is significantly larger than the near point K for all the systems of multilayer MoS_2_. The smallest direct energy gap decreases and the interlayer coupling increases as the number of layers grow. With the layer number increases, a very small decrease of direct energy gap at point K and a more significant decrease of direct energy gap at point Γ can be observed, as a result, a faint red shift in the threshold energy and a bright red shift in the end of both JDOS and ${\varepsilon}_2^{xx}\left(\omega \right)$ plateaus can also be found. For monolayer MoS_2_, the smallest ΔE(NC − NV) at point Γ is 2.75 eV which is larger than that at point B1 (or point B2) with a value around 2.57 eV. When it goes to multilayer and bulk MoS_2_, the strong interlayer coupling near point Γ makes the smallest ΔE(NC − NV) at Γ less than those at point B1 (or point B2). Hence, monolayer owns the longest plateau of JDOS, which is between 1.64 eV and 2.57 eV. The shortest plateau of JDOS (from 1.59 eV to 2.09 eV) is shown in the bulk.

As the energy is increased to the value larger than the endpoint of long platform of the dielectric function, a peak A can be found at the position around 2.8 eV, for almost all the studied materials (Fig. 6a). The width of peak A for monolayer is narrower compared with those of multilayer MoS_2_; however, the intensity of peak A for monolayer is found to be a little stronger than multilayers. The differences between the imaginary parts of dielectric function for the monolayer and multilayer MoS_2_ are evident, on the other hand, the differences are small for multilayer MoS_2_.

In order to explore the detailed optical spectra of MoS_2_ multilayers, the real parts of the dielectric function *ε*_1_(*ω*), the absorption coefficients *α*(*ω*), and the reflectivity spectra *R*(*ω*) are presented in Fig. 6b–d. Our calculated data of bulk MoS_2_ for the real and imaginary parts of the dielectric function, *ε*_1_(*ω*) and *ε*_2_(*ω*), the absorption coefficient *α*(*ω*) and the reflectivity *R*(*ω*) agree well with the experimental data, except for the excitonic features near the band edge [48–50]. The calculated values of , which is called the static dielectric constant, for MoS_2_ multilayers and bulk can be found in Table 1. From Table 1, the calculated values of ${\varepsilon}_1^{xx}(0)$ for multilayers and bulk MoS_2_ are all around 15.5, which is very close to the experimental value of 15.0 for bulk MoS_2_ [50]. The values of ${\varepsilon}_1^{xx}(0)$ increase with the increasing number of MoS_2_ layers. For monolayer MoS_2_, a clear peak B of ${\varepsilon}_1^{xx}\left(\omega \right)$ appears about 2.54 eV. Peak B of monolayer is clearly more significant than multilayers, and they are all similar for multilayer MoS_2_. As the layer number increases, the sharp structures (peak B) also move left slightly. In Fig. 6c, we also observe the emergence of long plateaus in the absorption coefficients, and absorption coefficients are around 1.5 × 10^5^ cm^−1^ at the long plateaus. There are also small steps around the thresholds for the absorption coefficients, which are consistent to those of the imaginary parts of dielectric function. With the layer number increases, the threshold energy of absorption coefficient decreases, while the number of small steps increases at the starting point of the plateau. For monolayer and multilayer MoS_2_, strong absorption peaks emerge at visible light range (1.65–3.26 eV), and the monolayer MoS_2_ own a highest absorption coefficient of 1.3 × 10^6^ cm^−1^. The theoretical absorption coefficients for different number of MoS_2_ layers are compared with confocal absorption spectral imaging of MoS_2_ (the inset) [46], as shown in Fig. 6c. For monolayer and multilayer MoS_2_, a large peak of *α*(*ω*) can be found at the position around 2.8 eV for both the calculation and experiment [46, 51]. Furthermore, a smoothly increase of *α*(*ω*) can be found between 2.2 and 2.8 eV for both the theoretical and experimental curves. Therefore, from Fig. 6c, the calculated absorption coefficients of MoS_2_ multilayers show fairly good agreement with the experimental data [46], except for the excitonic peaks. The reflectivity spectra are given in Fig. 6d. The reflectivity spectra of MoS_2_ multilayers are all about 0.35–0.36 when energy is zero, which means that MoS_2_ system can reflect about 35 to 36% of the incident light. In the region of visible light, the maximum reflectivity of monolayer MoS_2_ is 64%, while the maxima of multilayer and bulk MoS_2_ are all about 58%. Because of the behaviors discussed, MoS_2_ monolayer and multilayers are being considered for photovoltaic applications.

## Conclusions

In this study, by employing ab initio calculations, the electronic and optical properties of MoS_2_ multilayers are investigated. Compared to bulk MoS_2_, the covalency and ionicity of monolayer MoS_2_ are found to be stronger, which results from larger quantum confinement in the monolayer. With the increase of the layer number, quantum confinement and intra-layer interaction both decrease, meanwhile, the interlayer coupling increases, which result in the decrease of the band gap and the minimum direct energy gap. As the layer number becomes larger than two, the optical and electronic properties of MoS_2_ multilayers start to exhibit those of bulk. Band structures of multilayers and bulk show splitting of bands mainly around the Γ-point; however, splitting of bands in the vicinity of K point are tiny, owing to the small interlayer coupling at point K.

For optical properties, Van Hove singularities lead to the occurrence of long plateaus in both JDOS and ${\varepsilon}_2^{xx}\left(\omega \right)$. At the beginnings of these long plateaus, monolayer, bilayer, and trilayer structures appear one, two, and three small steps, respectively. With the layer number increases, the number of small steps increases and the width of the small steps decreases, leading to unobvious steps. A small red shift in the threshold energy and a noticeable red shift in the end of both JDOS and ${\varepsilon}_2^{xx}\left(\omega \right)$ plateaus are observed, since the increased number of layers leads to small changes in the direct energy gap near point K (weak interlayer coupling) and larger changes near point Γ (stronger interlayer coupling). Thus, the longest plateau and shortest plateau of JDOS are from the monolayer and bulk, respectively. Our results demonstrate that the differences between electronic and optical properties for monolayer and multilayer MoS_2_ are significant; however, the differences are not obvious between the multilayer MoS_2_. The present data can help understand the properties of different layers of MoS_2_, which should be important for developing optoelectronic devices.

## Additional file

Additional file 1: **Figure S1.** The electronic states of MoS_2_ multilayers are insensitive to the spin-polarized effect, due to the overlaps of spin-up and spin-down band structures for all the cases. (DOC 2814 kb)

## Data Availability

The datasets supporting the conclusions of this article are included within the article.

## Abbreviations

*Δ*E: The direct energy gap
1L: Monolayer MoS_2_
2L: Bilayer MoS_2_
3L: Trilayer MoS_2_
4L: Four-layer MoS_2_
5L: Five-layer MoS_2_
6L: Six-layer MoS_2_
BSE: Bethe-Salpeter equation
BZ: Brillouin zone
CBM: Conduction band minimum
GGA: Generalized gradient approximation
GW: Quasi-particle energy calculation
JDOS: Joint density of states
MoS_2_: Molybdenum disulfide
NC: The ordinal numbers of conduction band
NV: The ordinal numbers of valence band
PAW: Projector augmented wave
PBE: Perdew-Burke-Ernzerhof
VASP: Vienna ab initio simulation package
VBM: Valence band maximum
VHS: Van Hove singularity
