# Supplementary material for: Dependence of Electronic and Optical Properties of MoS2 Multilayers on the Interlayer Coupling and Van Hove Singularity
Source: Nanoscale Res Lett. 2019 Aug 19;14:288. doi: 10.1186/s11671-019-3105-9 (PMC6702492; doi:10.1186/s11671-019-3105-9)
Supplement: Supplementary file 1 — Figure S1. The electronic states of MoS2 multilayers are insensitive to the spin-polarized effect, due to the overlaps of spin-up and spin-down band structures for all the cases. (DOC 2814 kb) [file 11671_2019_3105_MOESM1_ESM.doc]

**Additional file 1**

The spin-polarization calculations are performed. The spin-up and spin-down band structures of MoS_2_ multilayers are shown here. For all the cases, spin-up and spin-down band structures overlap, indicating that the electronic states of MoS_2_ multilayers are insensitive to the spin-polarized effect.


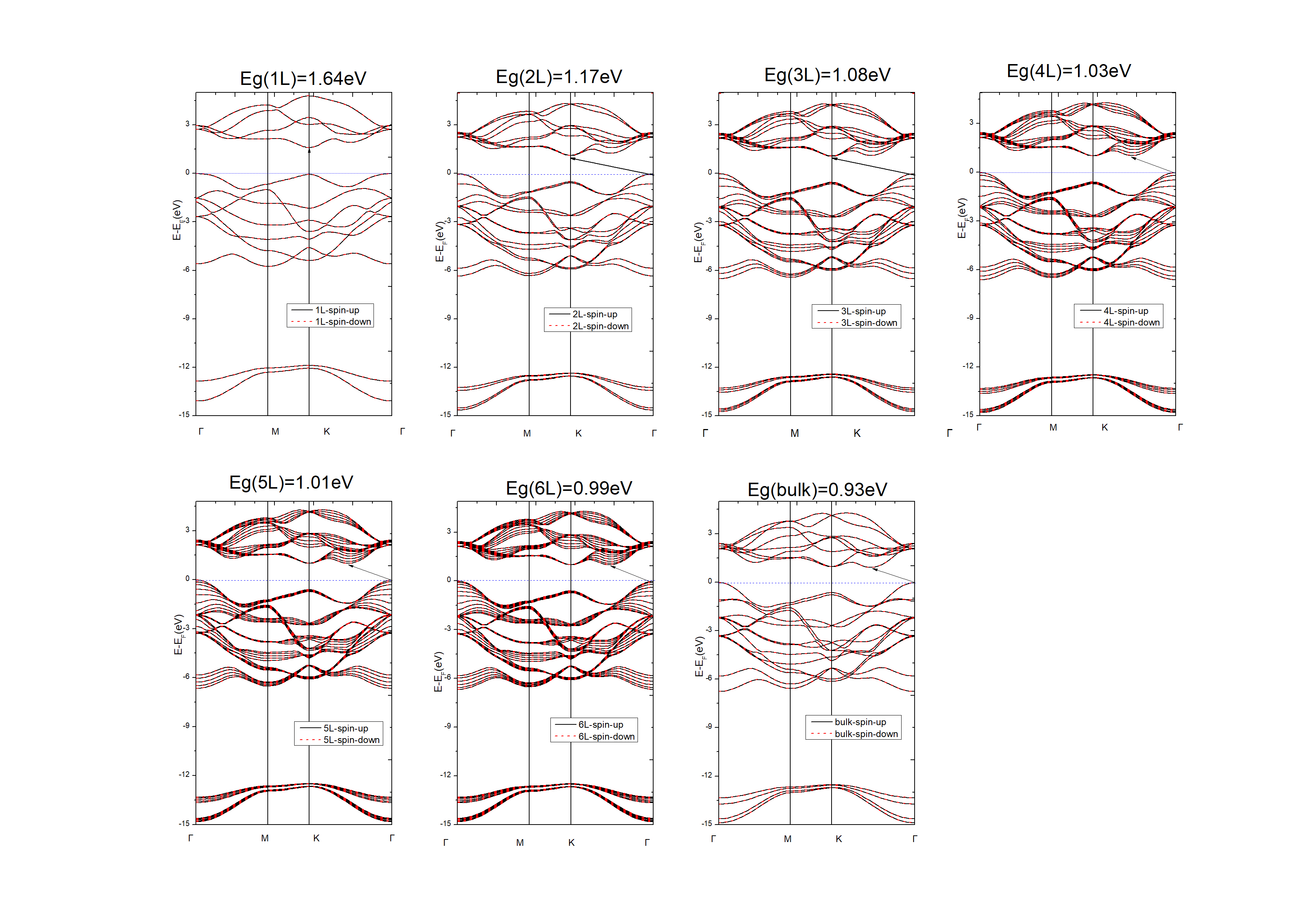


**Figure S1.** The electronic states of MoS_2_ multilayers are insensitive to the spin-polarized effect, due to the overlaps of spin-up and spin-down band structures for all the cases.
